# Angiotensin II and angiotensin-(1-7) decrease sFlt1 release in normal but not preeclamptic chorionic villi: an *in vitro *study

**DOI:** 10.1186/1477-7827-8-135

**Published:** 2010-11-04

**Authors:** Lauren Anton, David C Merrill, Liomar AA Neves, Courtney Gruver, Cheryl Moorefield, K Bridget Brosnihan

**Affiliations:** 1 Hypertension and Vascular Research Center, Wake Forest University School of Medicine, Winston-Salem, North Carolina, USA; 2 Department of Obstetrics and Gynecology, Wake Forest University School of Medicine, Winston-Salem, North Carolina, USA

## Abstract

**Background:**

During preeclampsia, placental angiogenesis is impaired. Factors released from the placenta including vascular endothelial growth factor (VEGF), placental growth factor (PLGF), soluble VEGF receptor 1 (sFlt1), and soluble endoglin (sEng) are regulatory molecules of placental development and function. While the renin angiotensin system has been shown to regulate angiogenic factors in other research fields, these mechanisms have not been extensively studied during pregnancy.

**Methods:**

We evaluated the effects of angiotensin II (Ang II) and angiotensin-(1-7) [Ang-(1-7)] on the release of VEGF, PLGF, sFlt1, and sEng from placental chorionic villi (CV). CV were collected from nulliparous third-trimester normotensive and preeclamptic subjects. CV were incubated for 0, 2, 4, and 16 hours with or without Ang II (1 nM and 1 microM) or Ang-(1-7) (1 nM and 1 microM). The release of VEGF, PLGF, sFlt1, sEng, lactate dehydrogenase (LDH), and human placenta lactogen (HPL) was measured by ELISA.

**Results:**

The release of sFlt1, PLGF, sEng from normal and preeclamptic CV increased over time. Release of sFlt1 and sEng was significantly higher from preeclamptic CV. VEGF was below the detectable level of the assay in normal and preeclamptic CV. After 2 hours, sFlt1 release from normal CV was significantly inhibited with Ang II (1 nM and 1 microM) and Ang-(1-7) (1 nM and 1 microM). There was a time-dependent increase in HPL indicating that the CV were functioning normally.

**Conclusions:**

Our study demonstrates a critical inhibitory role of angiotensin peptides on sFlt1 in normal pregnancy. Loss of this regulation in preeclampsia may allow sFlt1 to increase resulting in anti-angiogenesis and end organ damage in the mother.

## Background

Preeclampsia, clinically defined by the new onset of hypertension and proteinuria after 20 weeks gestation of pregnancy, affects 7-10% of pregnancies in the United Sates, 2-5% in European countries and over 10% in developing countries. Additionally, preeclampsia is responsible for large numbers of maternal and neonatal morbidity and mortality [1]. While the causes of preeclampsia remain unclear, significant advances have been made in understanding normal placental vasculogenesis and the pathophysiology of preeclampsia. It is thought that a balance of pro-angiogenic and anti-angiogenic factors modulate vessel growth in the placenta throughout pregnancy. Vascular endothelial growth factor (VEGF) and placental growth factor (PLGF) are pro-angiogenic molecules that act through two high affinity receptors, VEGF receptor 1 (VEGFR1 or Flt-1) and VEGF receptor 2 (VEGFR2 or KDR/Flk-1) [2,3]. In preeclampsia, the ischemic placenta has been shown to release factors, including the anti-angiogenic molecules soluble fms-like tyrosine kinase 1 (sFlt1) [4] and soluble endoglin (sEng) [5], that result in vasoconstriction and the end organ damage seen in the mother [6-8]. sFlt1, the soluble form of VEGFR1, can act as a decoy receptor by binding and sequestering circulating VEGF and PLGF thus preventing these molecules from binding to their active membrane bound endothelial cell receptors and consequently inhibiting angiogenesis [9]. Endoglin is a co-receptor for transforming growth factor β1 and β3 (TGF-β1 and TGF-β3) and has been shown to be expressed in endothelial cells and syncytiotrophoblasts [10-12]. sEng, a novel placenta derived soluble form of endoglin (Eng), is an anti-angiogenic protein that has been shown to inhibit TGF-β1 receptor binding leading to dysregulation of TGF-β1 signaling in the vasculature [5]. It has recently been shown that sFlt1 and sEng acting through different pathways work in concert to induce severe preeclampsia [5]. The molecular mechanisms that regulate sFlt1 release may also regulate sEng release from the preeclamptic placenta, however, the upstream regulators of these anti-angiogenic proteins remains unknown.

The RAS is an endocrine circulating system that is responsible for blood pressure, salt and fluid homeostasis. During normal pregnancy the RAS is activated due to increased estrogen levels which consequently cause levels of angiotensinogen [13,14] and renin [15-18] to rise. In association with the increases in angiotensinogen and renin, angiotensin II (Ang II) is up-regulated. The recently discovered peptide of the RAS, angiotensin-(1-7) [Ang-(1-7)] [19,20], has been shown in previous studies to increase in the circulation in the third trimester of pregnancy [21] and in urine throughout pregnancy [22]. A balance of the two biologically active peptides of the RAS, Ang II, a vasoconstrictor and angiogenic molecule, and Ang-(1-7), a vasodilator and anti-angiogenic molecule, may be essential for the maintenance of normal pregnancy. Previous studies done in our laboratory have investigated circulating and local RAS component expression in normal and preeclamptic women. While plasma angiotensin I (Ang I), Ang II, Ang-(1-7), and plasma renin activity are decreased in the circulation of preeclamptic women [21], in the chorionic villi of preeclamptic placentas, Ang II peptide levels and angiotensinogen and AT_1 _receptor mRNA levels were increased [23]. These findings in the chorionic villi of preeclamptic placentas lead us to hypothesize that Ang II and Ang-(1-7) might be regulators of sFlt1 and sEng release in preeclampsia. Although not much is known about the interaction between the RAS and angiogenic growth factors during pregnancy, Ang II has been shown to regulate angiogenesis through alterations in VEGF expression in the non-pregnant state [24-28]. Even less is known about the regulation of RAS peptides on the newly discovered modulators of placental angiogenesis such as sFlt1 and sEng. Therefore, in this study, we investigated the effect of Ang II and Ang-(1-7) on the release of sFlt1, sEng, VEGF, PLGF, and human placental lactogen (HPL) (functional control) from normal and preeclamptic chorionic villous explants.

## Methods

### Human subjects

These experiments were conducted using human placental tissue collected from woman with both normal pregnancies and preeclampsia. Placental tissue was collected from women who had either cesarean section or vaginal deliveries. Two separate groups of subjects were included. Group 1 (n = 21) consisted of normotensive pregnant subjects who have remained normotensive throughout pregnancy (systolic/diastolic BP < 140/90 mmHg), have no history of chronic blood pressure elevation, and have an absence of proteinuria. Group 2 (n = 15) consisted of preeclamptic subjects who developed new onset hypertension (systolic/diastolic BP > 140/90 mmHg) and proteinuria (1+ or equivalent to 30 mg/dl) proteinuria or ≥ 300 mg in 24 hour urine sample) after the 20^th ^week of gestation. Blood pressure readings are reported as the highest blood pressure measured in the labor and delivery suite prior to delivery. Subjects in the two groups were matched according to gestational age and parity (all nulliparous). Patients with evidence of chorioamnionitis were excluded. Women in both groups were over age 18 years and less than age 50 years and were free of other known cardiovascular, renal, connective tissue diseases, diabetes, cancer, or hyperplasia.

The study was approved by the institutional review boards (IRB) at both Wake Forest University School of Medicine and Forsyth Medical Center. The procedures followed were in accordance with institutional guidelines. After signed informed consent was obtained and the baby and placenta were delivered, placental samples were taken.

### Experimental procedures

For both normal pregnant and preeclamptic patients, immediately following delivery, the whole placenta was collected on ice and large tissue sections were taken from the center of the placenta, near the umbilical cord attachment site and at the periphery of the placenta. More specifically, five tissue sections were collected from each placenta. One piece of tissue was collected directly adjacent to the umbilical cord insertion site. The placenta was then divided into four quadrants where one piece of tissue from the periphery of the placenta was collected per quadrant. Areas of fibrotic lesions, heavy calcification and blood clotting were avoided. The total amount of time from delivery of the placenta until the samples were collected did not exceed fifteen minutes. The placental tissues were extensively washed in sterile phosphate buffered saline and placed on ice in a nutrient rich media containing high glucose Dulbecco's Modified Eagle's Medium (DMEM), 2.5% FBS, and antibiotics, for transport to the lab. The placental tissue sections were then dissected to isolate the chorionic villi in a serum free media made up of low glucose DMEM/F-12, non-essential amino acids (NEAA), glucose, sodium pyruvate, L-gluatamine, linoleic acid, NaHCO_3_, ex-cyte enhancement media supplement (Millipore, Billerica, MA), and antibiotics.

### Human placental chorionic villous explant culture

Chorionic villi (1.0 gram) dissected from human placental tissues taken from normal pregnant and preeclamptic women were placed into 25 ml Erlenmeyer flasks containing 10 ml of serum free media. The flasks were placed into a Dubnoff metabolic shaking bath (Precision Scientific, Winchester, VA) under constant shaking at 37°C in a humidified atmosphere of 5% N_2 _(pO_2 _in media = 60 mm Hg) supplemented with 5% CO_2 _[29]. Under normal physiological conditions, the oxygen tension within the intervillous space at term is ~50 to 60 mm Hg [30,31]. After 15 minutes of incubation, the amount of oxygen in the media was analyzed using a Rapidlab 248 blood gas machine (Bayer HealthCare, East Walpole, MA). The pO_2 _and pCO_2 _were monitored throughout the experiment to ensure a consistent gas environment. The tissue was cultured in the metabolic shaker for 2 hours as an equilibration period. The media was removed and 5 ml of fresh serum free media treated with or without 1 nM or 1 μM Ang II or Ang-(1-7) was added to the villous explants. In addition, normal and preeclamptic chorionic villi (n = 4) were incubated in serum free media plus cell lysis buffer in order to evoke a maximal amount of LDH release. Values were expressed as percent of maximal release. The chorionic villi were incubated for 0, 2, 4, or 16 hours. The media was collected and frozen at -80°C for further analysis. The chorionic villous tissue was weighed and a small piece was removed and placed in 4% formaldehyde for histological analysis.

### Enzyme linked immunoassay (ELISA)

VEGF, PLGF, sFlt1, sEng, and human placental lactogen (HPL) released into the cell culture media from normal pregnant and preeclamptic chorionic villi explants were measured by ligand specific commercially available ELISA kits that utilize a quantitative sandwich enzyme immunoassay technique using regents from R&D Systems (Minneapolis, MN) for VEGF, PLGF, and sFlt1, and sEng. For HPL, a kit from Alpco Diagnostics (Windham, NH) was used.

### Lactate dehydrogenase (LDH) in vitro toxicology assay

LDH released into the media from normal and preeclamptic chorionic villi throughout the chorionic villi explant experiment was measured by a commercially available LDH in vitro toxicology kit (Sigma, St. Louis, MS).

### Statistical analysis

The data were analyzed with a two way analysis of variance (ANOVA). When there was a significant interaction, subsequent analysis included the Bonferroni's post hoc test for multiple comparisons to determine the effects between time points and treatment groups. The Student's t test for unpaired observations was used for comparing two groups (GraphPad Software, San Diego, CA). All data were normally distributed. A p value of less than 0.05 was considered statistically different. All arithmetic means are presented ± standard deviation (SD).

## Results

### Clinical profile of normal and preeclamptic patients

Table 1 shows the clinical characteristics of the 21 women with normal pregnancies and the 15 women with preeclampsia for this study. The preeclamptic women had significant hypertension with increased systolic, diastolic, and mean blood pressures. In addition, the preeclamptic subjects showed proteinuria measured as greater than 1+ or equivalent to 30 mg/dl on a urine dipstick. The preeclamptic subjects also had a significantly lower birth weight than normal pregnant women. No significant differences were seen in maternal age or body mass index. The normal and preeclamptic subjects were matched for gestational age.

**Table 1:** Clinical profile of the study population

Patient clinical characteristics	Normal pregnancy	Preeclamptic pregnancy
*n*	21	15
Age (years)	26.3 ± 5.9	23.7 ± 4.5
Body Mass Index (BMI) (kg/m_2_)	29.8 ± 6.3	29.6 ± 4.6
Birth weight (grams)	3035.4 ± 535.0	2599 ± 697.3^a^
Gestational age(weeks)	37.8 ± 2.1	s36.6 ± 2.4
Systolic blood pressure (mm Hg)	135 ± 13	174 ± 15^b^
Diastolic blood pressure (mm Hg)	77 ± 9	110 ± 7^b^
Mean blood pressure (mm Hg)	96 ± 9	131 ± 8^b^
Proteinuria (0-4+)	none	>1+
Number of patients receiving anti-hypertensive medications	none	5

Values are expressed as mean ± SD. ^a^p < 0.05, ^b^p < 0.0001 versus normal pregnancy

^‡^Anti-hypertensive treatment for five preeclamptic patients included Hydralazine and Labetalol.

### Time dependent release of sFlt1, sEng, PLGF, VEGF, and HPL from normal and preeclamptic chorionic villi explants

**Figure 1:**
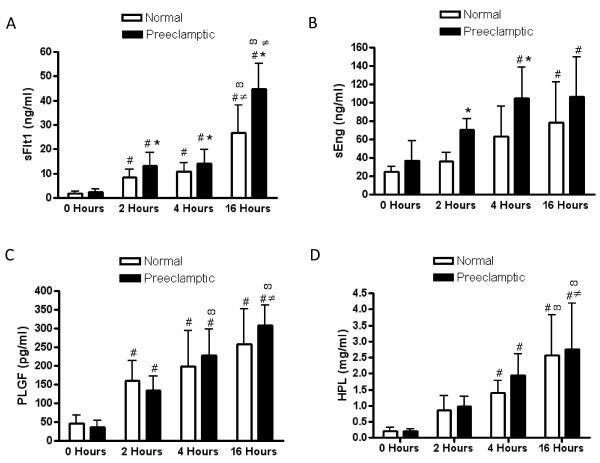
**Time dependent release of sFlt1, sEng, PLGF, and HPL**. sFlt1 (**A**), sEng (**B**), PLGF (**C**), and HPL (**D**) release were measured in the media after 0, 2, 4, and 16 hours of incubation in normal (n = 21) and preeclamptic (n = 15) chorionic villous explants. Data are expressed as the mean ± SD. * p < 0.05 preeclamptic versus normal chorionic villi, # p < 0.05 relative to 0 hours, ∞ p < 0.05 relative to 2 hours, and ≠ p < 0.05 relative to 4 hours in either normal or preeclamptic chorionic villi explants.

The time dependent release of sFlt1, sEng, PLGF, and HPL from normal and preeclamptic chorionic villi explants was measured at 0, 2, 4, and 16 hours of incubation as shown in Figure 1. The concentration of sFlt1 was significantly increased over time (p < 0.0001) at 2 (p < 0.05), 4 (p < 0.01), and 16 (p < 0.001) hours in both normal and preeclamptic chorionic villi. In addition, sFlt1 at 16 hours was significantly different from 2 (p < 0.001) and 4 (p < 0.001) hours in normal and preeclamptic chorionic villi. The concentration of sFlt1 was significantly higher in the preeclamptic subjects (p < 0.0001) at 2 (p < 0.05), 4 (p < 0.05), and 16 (p < 0.05) hours (Figure 1A).

The concentration of sEng was significantly increased over time (p < 0.0001) in normal and preeclamptic chorionic villi. sEng was significantly increased from baseline (0 hours) at 4 (p < 0.01) and 16 (p < 0.01) hours in preeclamptic chorionic villi and only at 16 hours (p < 0.05) in normal chorionic villi. In addition, sEng was significantly higher (p < 0.01) in preeclamptic explants when compared to normal pregnant women at 2 (p < 0.01) and 4 (p < 0.05) hours (Figure 1B).

The concentration of PLGF from normal and preeclamptic chorionic villi was significantly increased over time (p < 0.0001) at 2 (p < 0.01), 4 (p < 0.001), and 16 (p < 0.001) hours. In addition, PLGF at 16 hours was significantly different from 2 (p < 0.001) and 4 (p < 0.05) hours only in the preeclamptic chorionic villi. No differences in PLGF were seen between normal and preeclamptic chorionic villi at each time point (Figure 1C).

VEGF from normal and preeclamptic chorionic villi was found to be below the detectable level of the assay at all time points (0-16 hours) (data not shown).

In these experiments, we measured the release of HPL from the chorionic villi as a positive control of tissue function to ensure that the chorionic villi remained intact and continued to produce HPL over time. The concentration of HPL was significantly increased over time from 0 to 16 hours in both normal and preeclamptic chorionic villi (p < 0.0001). HPL was significantly increased from baseline (0 hours) at 4 (p < 0.01) and 16 (p < 0.01) hours in normal and preeclamptic chorionic villi. HPL at 16 hours was significantly different from both 2 (p < 0.001) and 4 (p < 0.05) hours in the preeclamptic chorionic villi. In normal chorionic villi, the concentration of HPL at 16 hours was significantly different after 4 hours (p < 0.01) of incubation. No significant differences in HPL were seen between normal and preeclamptic chorionic villi at each time point (Figure 1D).

In order to monitor the integrity of the chorionic villi, LDH was measured as an index of chorionic villi degradation and breakdown throughout the time course of the experiment. Maximal release of LDH (1090 ± 156 U/L) was elicited by incubating the chorionic villi with cell lysis buffer. The basal concentration of LDH in the media at 2 and 4 hours was only 5% and 7% of maximal release, respectively and not significantly different from the 0 time point. At 16 hours, LDH levels were less than 15% of the maximal cell lysis buffer evoked LDH. There was no difference in LDH concentration between normal and preeclamptic chorionic villi except for a slightly higher level at 2 hours in the preeclamptic samples (data not shown). Additionally, investigation of the chorionic villi histology after 16 hours of incubation showed no changes in tissue morphology or evidence of tissue damage (data not shown).

### The effect of Ang II and Ang-(1-7) on the release of sFlt1 in normal and preeclamptic chorionic villi

**Figure 2:**
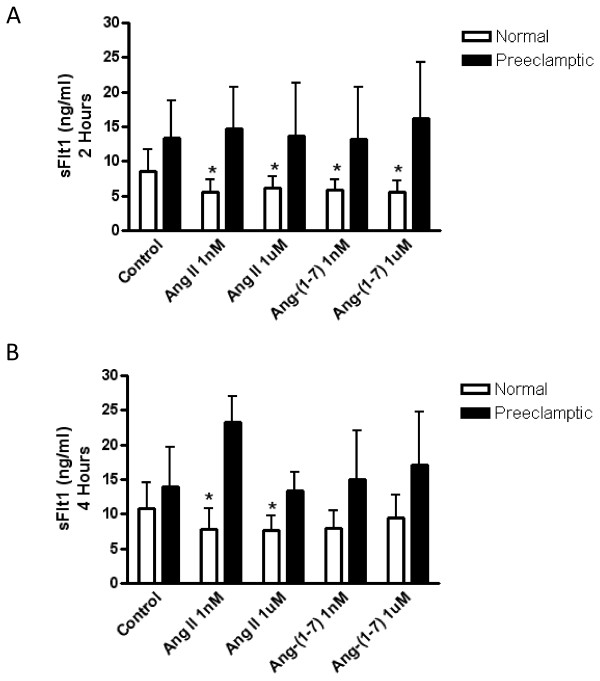
**The effects of Ang II and Ang-(1-7) on sFlt1 release at 2 hours and 4 hours of incubation**. Normal (n = 21) and preeclamptic (n = 15) chorionic villi were incubated with or without Ang II (1 nM and 1 μM) or Ang-(1-7) (1 nM and 1 μM) and sFlt1 release was measured by ELISA after 2 hours (**A**) and 4 hours (**B**) of incubation. Data are expressed as the mean ± SD. * p < 0.05 versus control of normal chorionic villi.

Normal and preeclamptic chorionic villi were treated with Ang II and Ang-(1-7) (1 nM and 1 μM) for 0, 2, 4, and 16 hours and the amount of sFlt1 released into the media was measured. As seen in Figure 2A, after 2 hours of incubation, sFlt1 release was significantly inhibited by treatment with 1 nM Ang II (p < 0.01), 1 μM Ang II (p < 0.05), 1 nM Ang-(1-7) (p < 0.01), and 1 μM Ang-(1-7) (p < 0.01) in the chorionic villi from normal pregnant women. After 4 hours of incubation, sFlt1 release is still significantly inhibited by 1 nM Ang II (p < 0.05) and 1 μM Ang II (p < 0.05) in the normal chorionic villi (Figure 2B). However, treatment with Ang-(1-7) no longer significantly inhibits sFlt1 release from normal chorionic villi. Ang II and Ang-(1-7) had no effect on sFlt1 release in the preeclamptic chorionic villi at any time point.

### The effect of Ang II and Ang-(1-7) on the release of PLGF and sEng in normal and preeclamptic chorionic villi

**Figure 3:**
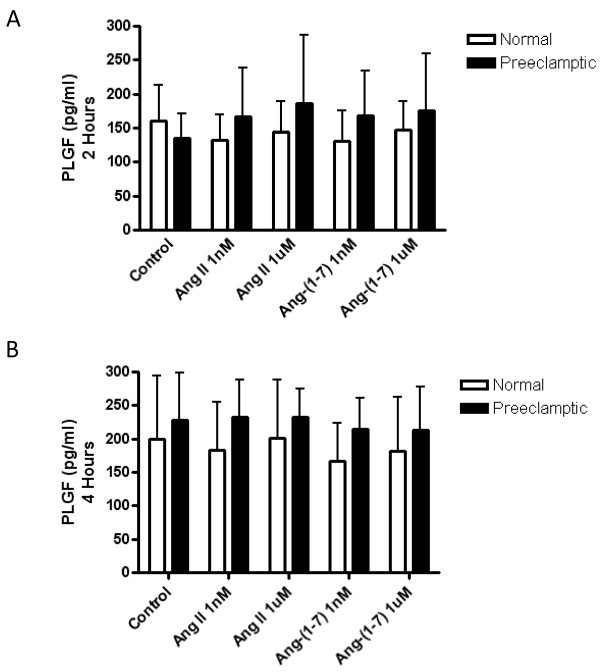
**The effects of Ang II and Ang-(1-7) on PLGF release at 2 hours and 4 hours of incubation**. Normal (n = 9) and preeclamptic (n = 15) chorionic villi were incubated with or without Ang II (1 nM and 1 μM) or Ang-(1-7) (1 nM and 1 μM) and PLGF release was measured by ELISA after 2 hours (**A**) and 4 hours (**B**) of incubation. Data are expressed as the mean ± SD.

Normal and preeclamptic chorionic villi were treated with Ang II and Ang-(1-7) (1 nM and 1 μM) for 0, 2, 4, and 16 hours and the amount of PLGF and sEng released into the media was measured. PLGF (Figure 3A and 3B) and sEng (data not shown) released from normal and preeclamptic chorionic villi was unchanged after Ang II (1 nM and 1 μM) or Ang-(1-7) (1 nM and 1 μM) treatment for 2 and 4 hours. In addition, no significant changes were seen in PLGF or sEng release by Ang II (1 nM and 1 μM) or Ang-(1-7) (1 nM and 1 μM) treatment in normal or preeclamptic chorionic villi at 16 hours (data not shown).

## Discussion

This study demonstrates that sFlt1, sEng, PLGF, and HPL are released from the chorionic villi of both normal and preeclamptic women and that the release of sFlt1 is regulated by Ang II and Ang-(1-7). We found that sFlt1 and sEng release from the chorionic villi is higher in preeclamptic women when compared to normal. Both Ang II and Ang-(1-7) decreased the release of sFlt1 from the chorionic villi of normal pregnant women but not from women with preeclampsia after two hours of incubation. In addition, we found that sEng, PLGF, and HPL release from both normal and preeclamptic chorionic villi was unchanged with Ang II and Ang-(1-7) treatment. This study provides evidence that the biologically active renin angiotensin system peptides, Ang II and Ang-(1-7), are involved in regulating the release of the anti-angiogenic growth factor, sFlt1, from the chorionic villi in normal but not preeclamptic pregnancies.

### Ang II and Ang-(1-7) decreased sFlt1 release from normal chorionic villous explants

In normal chorionic villi, sFlt1 was significantly decreased by both Ang II and Ang-(1-7) after 2 hours of incubation. While Ang II continued to cause a significant decrease in sFlt1 release after 4 hours of treatment, there was no longer a significant Ang-(1-7) mediated effect. By 16 hours there was no significant effect on sFlt1 release by Ang II or Ang-(1-7). A previous study investigated the effects of Ang II on sFlt1 release in normal human placental villous explants and found that Ang II treatment after 48 and 72 hours increased sFlt1 release [32]. The discrepancies in the findings between the two studies may be explained by the differences in experimental length. While the human placental villous explant studies done by Zhou et al. [32] involved Ang II treatment over a period of 48 and 72 hours, our study was never longer than 16 hours and showed significant effects of Ang II on sFlt1 release as early as two hours. Therefore, the regulation of sFlt1 release by Ang II may be critically time dependent.

We monitored the stability of the chorionic villous tissue throughout the experiment. Basal levels of LDH release never reached higher than 5-7% of the cell lysis evoked LDH levels at the early time points of incubation (2 and 4 hours) where the observed effects of Ang II and Ang-(1-7) on sFlt1 release were seen. The study by Zhou et al [32] did not provide any measurements of the stability of the placental villous explants at the 48 and 72 hour time points. LDH levels measured in our chorionic villi explants incubated for 48 and 72 hours were greater than 20% of the maximal LDH release (data not shown). This may indicate that the increased sFlt1 release shown by Zhou et al. [32] could be associated with tissue damage from the longer incubation time and could contribute to the differences between the findings. Investigation of the histology of the chorionic villi in both normal and preeclamptic subjects revealed no damage or abnormalities to the tissue during the 16 hours of incubation (data not shown). HPL, a measurement of tissue function, showed an increasing trend at 2 hours and a significant increase at 4 hours. Therefore, the decrease in sFlt1 release by Ang II and Ang-(1-7) in our study occurs when there is no evidence of tissue breakdown and clear evidence of tissue integrity by the release of HPL.

The Ang II-mediated decrease in sFlt1 release in normal chorionic villi is further corroborated by previous studies which have shown that Ang II initiates angiogenesis by decreasing sFlt1 release and consequently causing an increase in free VEGF. Both Ang II and Ang-(1-7) have been shown to act as regulators of the angiogenic process through alterations in VEGF production. Ang II, acting through the AT_1 _receptor, can induce the over expression of VEGF causing an increase in angiogenesis [24-28]. Interestingly, we have previously shown that the AT_1 _receptor is the predominant angiotensin receptor subtype present in the chorionic villi of the placenta by both real time RT-PCR and *in vitro *receptor autoradiography [23] indicating that Ang II would most likely be acting through the AT_1 _receptor to decrease sFlt1 release. Therefore, it can be hypothesized that during normal pregnancy Ang II decreases sFlt1 release allowing unbound VEGF and PLGF to initiate the angiogenic process and contribute to endothelial function, a potentially beneficial effect for normal pregnancy.

The decrease in sFlt1 release after treatment with Ang-(1-7) (1 nM and 1 μM) in normal chorionic villi was unexpected since previous studies have shown that Ang-(1-7) acts primarily as an anti-angiogenic protein. Our laboratory has shown that Ang-(1-7) inhibits *in vitro *tube formation in human umbilical vein endothelial cells (HUVEC) [33]. Ang-(1-7) also inhibits angiogenesis and fibrovascular tissue growth in cultured rat thoracic aortic vascular smooth muscle cells and in a mouse sponge model of angiogenesis [34,35]. More recent studies have shown that Ang-(1-7) inhibits lung tumor formation and growth [36]. The specific effects of Ang-(1-7) during pregnancy have not been extensively studied so the role of Ang-(1-7) in the angiogenic process in the placenta is unknown. The finding that both Ang II and Ang-(1-7) inhibited the release of sFlt1 is unusual since their actions are in most instances opposite. However, an early study reported that Ang II and Ang-(1-7) have similar effects by increasing arginine vasopressin release from rat hypothalmic explants [37]. There is a possibility that the high dose (1 μM) of Ang-(1-7) could be acting at the AT_1 _receptor and could; therefore, be causing Ang II-like actions of decreased sFlt1 release. However, activation of the AT_1 _receptor at a lower dose (1 nM) of Ang-(1-7) would be unlikely. It has been previously shown that Ang-(1-7) has a poor affinity for the AT_1 _receptor [38,39]. Therefore, this would suggest that Ang-(1-7) is acting at its own specific AT_1-7 _receptor. The fact that Ang II and Ang-(1-7) both decrease sFlt1 release in normal chorionic villi does provide strong evidence that the RAS is an important mediator of angiogenesis in the placenta.

### Loss of Ang II and Ang-(1-7) regulation on sFlt1 release from preeclamptic chorionic villi

Investigation of sFlt1 in preeclamptic chorionic villi showed a significant increase in release when compared to normal chorionic villi over the time course of the experiment. Our results agree with several previous studies where sFlt1 protein levels were found to be up-regulated in the placenta of women with preeclampsia [4,40,41]. No changes in sFlt1 release from preeclamptic chorionic villi were seen with treatment of either Ang II or Ang-(1-7). This result indicates that the regulation seen with Ang II and Ang-(1-7) on sFlt1 release in the normal chorionic villi is lost in women with preeclampsia. The loss of sFlt1 regulation in the preeclamptic placenta provides evidence for the fact that sFlt1 would be released into the circulation uninhibited in the presence of Ang II and Ang-(1-7), where it can bind to both VEGF and PLGF thus blocking them from binding to their membrane bound receptors and preventing the downstream signaling cascades needed to initiate angiogenesis. The decrease in angiogenesis caused by the loss of Ang II and Ang-(1-7) regulation within the placenta could be contributing to the placental ischemia seen in women with preeclampsia.

### sEng release was unchanged by Ang II and Ang-(1-7) in normal and preeclamptic chorionic villi

Along with sFlt1, we investigated the effects of Ang II and Ang-(1-7) treatment on sEng release from normal and preeclamptic chorionic villi. We found a significant increase in sEng release from preeclamptic chorionic villi when compared to normal at the 2 hour and 4 hour time points. In agreement with our results, sEng has been shown in previous studies to be up-regulated in the placenta of preeclamptic women [5]. No changes in sEng release were seen with Ang II and Ang-(1-7) treatment in normal and preeclamptic chorionic villi. The fact that sEng release was not changed with either Ang II or Ang-(1-7) treatment, while both peptides had an effect on sFlt1 release indicates that the renin angiotensin system may play a more predominant role in regulating angiogenesis through sFlt1 rather than sEng. Even though sFlt1 and sEng act in concert to control the mechanisms that may be responsible for the development of preeclampsia, each of these anti-angiogenic molecules may be regulated differently. Therefore, additional studies investigating the upstream molecular regulators of sEng are warranted.

### VEGF and PLGF release were unchanged by Ang II and Ang-(1-7) in normal and preeclamptic chorionic villi

In addition to sFlt1 and sEng release, we investigated VEGF and PLGF release from normal and preeclamptic chorionic villi. VEGF release was found to be below the detectable level of the assay in both normal and preeclamptic chorionic villi at all time points measured. While circulating VEGF levels have been measurable in the serum of normal and preeclamptic women, they were found to be very low [4,40]. Contrary to our results, a previous study using human placental explants was able to measure VEGF release after 24 hours of incubation [29]. These conflicting results could be due to the fact that the study done by Ahmed and Ahmed [29] incubated their tissues in a smaller amount of media allowing the concentration of VEGF release to be measurable. The very low amounts of VEGF released by the chorionic villi indicate that PLGF may be the more predominant pro-angiogenic growth factor in the placental chorionic villi during pregnancy. In this study we found that there was a trend for a decrease in PLGF release in the control (non-treated) preeclamptic chorionic villi when compared to normal at the two hour time point, however it did not reach statistical significance. These results agree with previous studies that have shown that PLGF release is decreased in association with increases in sFlt1 in preeclamptic women [4,40,42]. Treatment of normal and preeclamptic chorionic villi with Ang II and Ang-(1-7) had no effects on PLGF release. No previous studies have been done to investigate the effects of Ang II on PLGF. Although our results show that Ang II and Ang-(1-7) did not have an effect on PLGF regulation in the chorionic villi, the presence of PLGF in this tissue indicates that this angiogenic factor could be playing an important role in balancing the anti-angiogenic effects of sFlt1 during normal pregnancy. In women with preeclampsia, where sFlt1 release is significantly higher and PLGF release is unchanged or lower, the angiogenic balance would be tilted towards anti-angiogenesis resulting in decreased blood vessel growth within the placenta.

Interestingly, the concentrations of sFlt1 and sEng release were about 1000 times higher than PLGF release in both normal and preeclamptic chorionic villi. This indicates that sFlt1 and sEng may be the predominant factors in angiogenesis regulation within the placenta. However, this does not rule out an effect of PLGF release on the angiogenic balance in normal and preeclamptic patients. In normal pregnancy, the decrease in sFlt1 release from the chorionic villi by Ang II and Ang-(1-7) would be consistent with increased levels of free VEGF and PLGF and their subsequent effects on angiogenesis.

## Conclusions

This study provides evidence that the RAS components, Ang II and Ang-(1-7), play a role in controlling the release of angiogenic growth factors from chorionic villi. Both Ang II and Ang-(1-7) decrease sFlt1 release in normal chorionic villi but not in the chorionic villi from preeclamptic women. Because the regulation of sFlt1 is critical to the process of angiogenesis, our data suggest that the down-regulation of sFlt1 release by Ang II and Ang-(1-7) in normal chorionic villi may decrease the amount of sFlt1 available to bind to VEGF and PLGF and promote their actions on angiogenesis. In women with preeclampsia, where we have shown Ang II levels to be increased in the chorionic villi [23], the regulation of Ang II on sFlt1 is lost causing the release of sFlt1 to be uninhibited contributing to an enhanced state of anti-angiogenesis.

## Competing interests

The authors declare that they have no competing interests.

## Authors' contributions

LA contributed to the development of the study design, performed all elements of the laboratory based studies and was primarily responsible for drafting this manuscript. DCM is the primary physician overseeing this study and was instrumental in obtaining the patient samples needed to carry out these studies. LAAN participated in the study design and helped to put together the protocol for the chorionic villous explant experiments. CG and CM are research nurses who identified and consented all patients involved in this study. KBB is the primary investigator in the laboratory and was responsible for designing the study, overseeing all experiments and research progress, and contributed significantly to this manuscript. All authors have read and approved the final manuscript.
